# Supplementary material for: The combination of genomic offset and niche modelling provides insights into climate change-driven vulnerability
Source: Nat Commun. 2022 Aug 16;13:4821. doi: 10.1038/s41467-022-32546-z (PMC9381542; doi:10.1038/s41467-022-32546-z)
Supplement: Supplementary file 1 — Supplementary figures and tables [file 41467_2022_32546_MOESM1_ESM.pdf]

Supplementary Information

The combination of genomic offset and niche modelling provides insights into climate change-driven vulnerability

Chen et al.

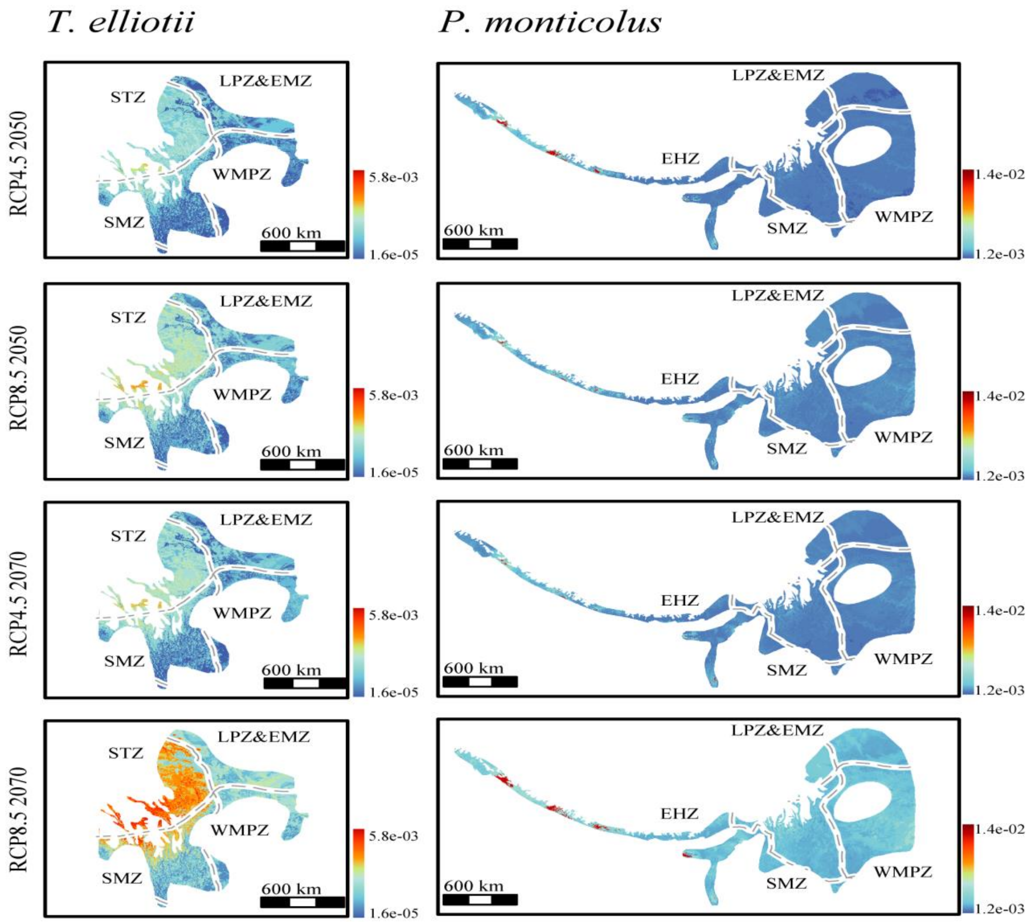

Supplementary Fig. 1. Genomic offsets to the future climate change. Left, *T. elliotii*; right, *P. monticolus*. For the future climate projections, we used moderate scenario (representative concentration pathway, RCP 4.5W/M^2) and the worst scenario (RCP 8.5 W/M^2) of greenhouse gas emission trajectories at the 2050 and 2070 horizons, respectively. These results suggest a magnitude-dependent genomic offset.

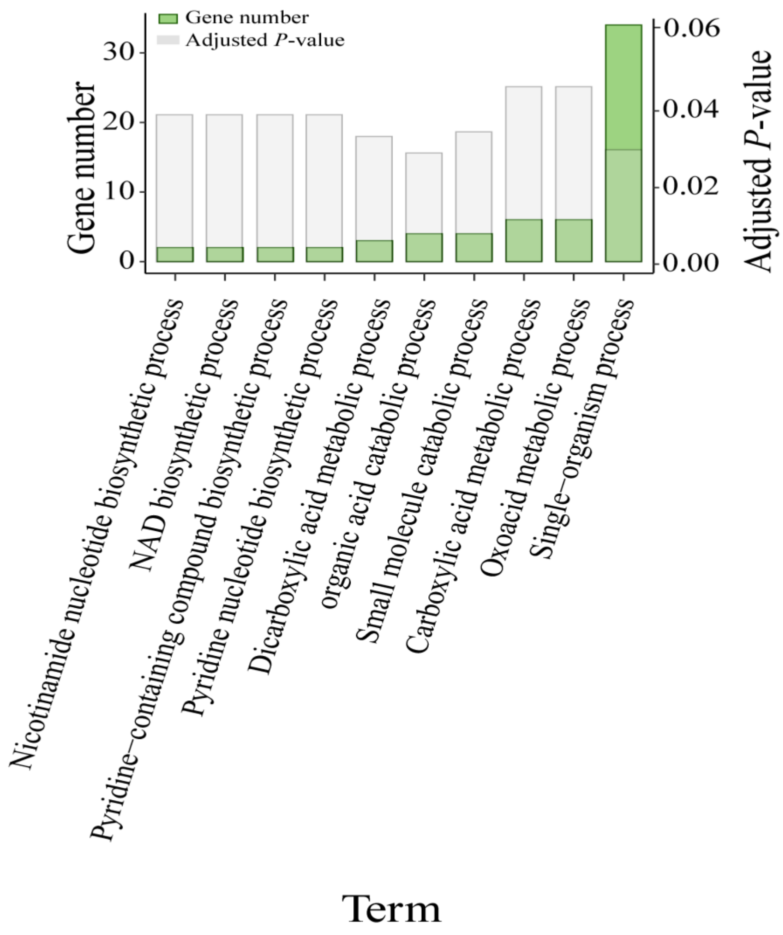

Supplementary Fig. 2. The SNPs significantly associated with the top climatic variables are enriched in the genes that are functionally related to the catalytic and metabolic processes. Only result from *P. monticolus* is shown, as 23 genes identified in *T. elliotii* did not enrich any significant GO term or pathway after multiple correction of *P* value.

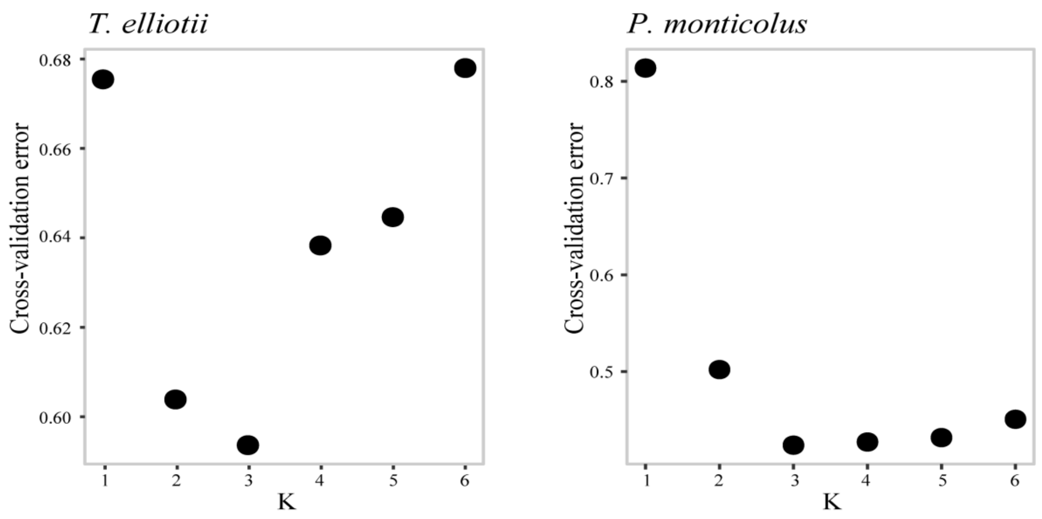

Supplementary Fig. 3. Cross-validation error for the coancestry cluster between 1 and 6 in Admixture 1.3. The optimal cluster (*K*=3) was identified by the smallest cross-validation error.

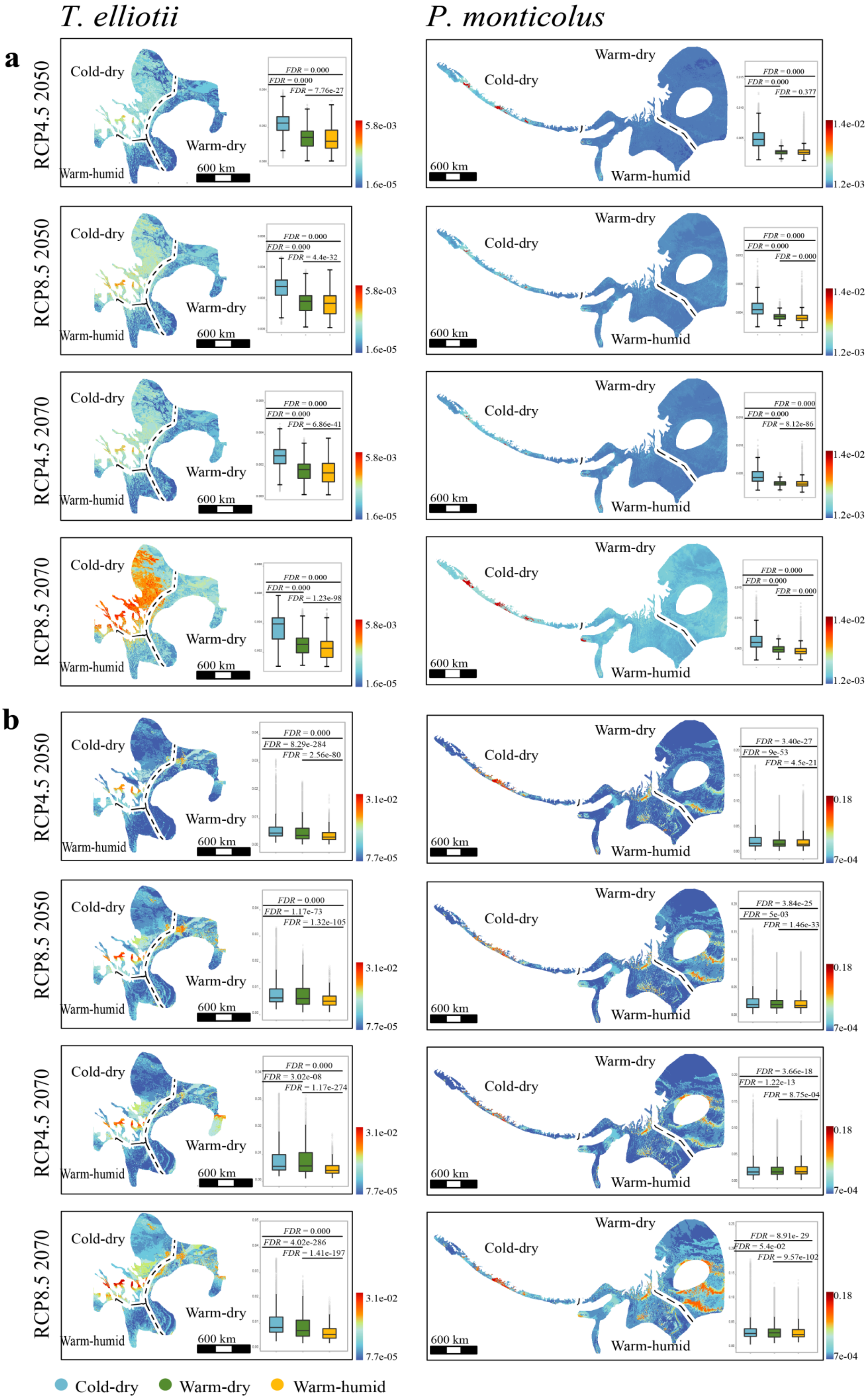
T. elliotii
P. monticolus
a
b
RCP4.5 2050
RCP8.5 2050
RCP4.5 2070
RCP8.5 2070
Cold-dry
Warm-dry
Warm-humid
600 km
5.8e-03
1.6e-05
1.4e-02
1.2e-03
3.1e-02
7.7e-05
0.18
7e-04
FDR = 0.000
FDR = 7.76e-27
FDR = 0.377
FDR = 4.4e-32
FDR = 6.86e-41
FDR = 8.12e-86
FDR = 1.23e-98
FDR = 8.29e-284
FDR = 2.56e-80
FDR = 3.40e-27
FDR = 9e-53
FDR = 4.5e-21
FDR = 1.17e-73
FDR = 1.32e-105
FDR = 3.84e-25
FDR = 5e-03
FDR = 1.46e-33
FDR = 3.02e-08
FDR = 1.17e-274
FDR = 3.66e-18
FDR = 1.22e-13
FDR = 8.75e-04
FDR = 4.02e-286
FDR = 1.41e-197
FDR = 8.91e- 29
FDR = 5.4e-02
FDR = 9.57e-102
Cold-dry
Warm-dry
Warm-humid

Supplementary Fig. 4. GradientForest predicted genomic offsets under different future climatic conditions (RCP4.5 2050, RCP8.5 2050, RCP4.5 2070 and PCP8.5 2070) in the *T. elliotii* and *P. monticolus*. (a) R^2 positive SNPs, (b) outlier SNPs. Inset, the cold-dry tolerant groups (*T. elliotii*, *n*=21,402 grids; *P. monticolus*, *n*=6,926 grids) show greater genomic offsets than the warm-humid (*T. elliotii*, *n*=7,061 grids; *P. monticolus*, *n*=30,336 grids) and warm-dry tolerant groups (*T. elliotii*, *n*=15,416 grids; *P. monticolus*, *n*=42,929 grids) in *T. elliotii* (left) and *P. monticolus* (right). We tested genomic offsets among these groups using the two-tailed Wilcoxon rank sum test and FDR correction for multiple comparisons. Note that spatial scales of the Y axis (genomic offsets) for the R^2 positive SNPs (a) differ from those for the outlier SNPs (b). The box plots show the median (center line) and 25th-75th percentiles (box limits). The whiskers extend to the top/bottom to the maxima and minima. Data beyond the end of the whiskers are considered outliers.

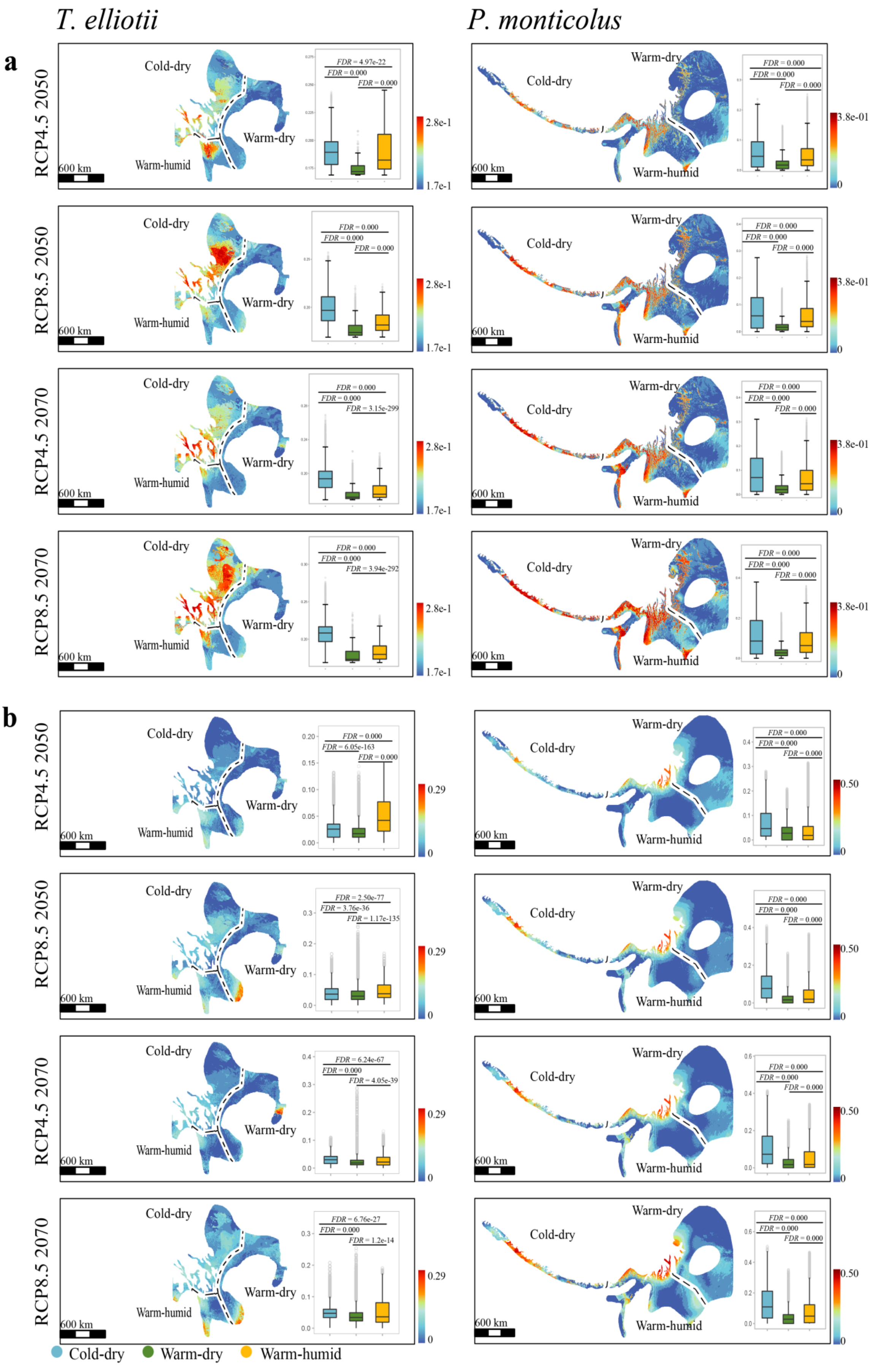
T. elliotii
P. monticolus
a
b
RCP4.5 2050
RCP8.5 2050
RCP4.5 2070
RCP8.5 2070
Cold-dry
Warm-dry
Warm-humid
600 km
2.8e-1
1.7e-1
3.8e-01
0
0.29
0.50
Cold-dry
Warm-dry
Warm-humid

Supplementary Fig. 5. Genomic offset analysis using generalized dissimilarity modelling (GDM). The predicted genomic offsets to future climatic conditions (RCP4.5 2050, RCP8.5 2050, RCP4.5 2070 and RCP8.5 2070). (a) R^2 positive SNPs, (b) outlier SNPs. Inset, the cold-dry tolerant groups (*T. elliotii*, *n*=21,402 grids; *P. monticolus*, *n*=6,926 grids) show greater genomic offsets than the warm-humid (*T. elliotii*, *n*=7,061 grids; *P. monticolus*, *n*=30,336 grids) and warm-dry tolerant groups (*T. elliotii*, *n*=15,416 grids; *P. monticolus*, *n*=42,929 grids) in *T. elliotii* (left) and *P. monticolus* (right). We tested genomic offsets among these groups using the two-tailed Wilcoxon rank sum test and FDR correction for multiple comparisons. Note that spatial scales of the Y axis (genomic offsets) for the R^2 positive SNPs (a) differ from those for the outlier SNPs (b). The box plots show the median (center line) and 25th-75th percentiles (box limits). The whiskers extend to the top/bottom to the maxima and minima. Data beyond the end of the whiskers are considered outliers.

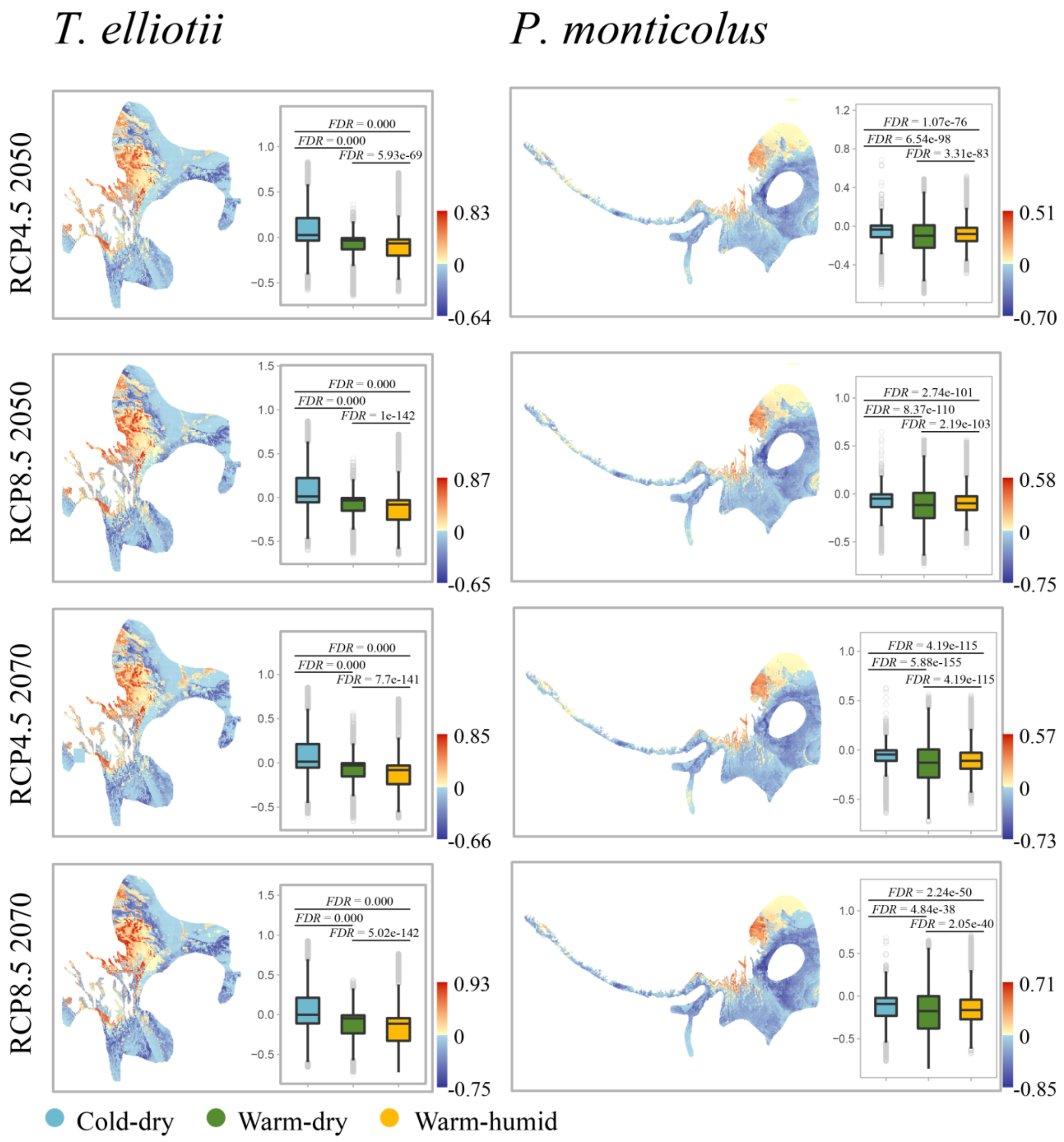

Supplementary Fig. 6. Niche suitability change between current and future climate (RCP4.5 2050, RCP8.5 2050, RCP4.5 2070 and RCP8.5 2070). The reddish colours show areas with increasing niche suitability (>0) and blue colours show the areas with decreasing niche suitability (<0). Inset, the warm-humid (*T. elliotii*, *n*=7,061 grids; *P. monticolus*, *n*=30,336 grids) and warm-dry tolerant groups (*T. elliotii*, *n*=15,416 grids; *P. monticolus*, *n*=42,929 grids) are under the severer challenge for niche suitability decline than are the cold-dry tolerant groups (*T. elliotii*, *n*=21,402 grids; *P. monticolus*, *n*=6,926 grids). We tested niche suitability change among these groups using the two-tailed Wilcoxon rank sum test and FDR correction for multiple comparisons. The box plots show the median (center line) and 25th-75th percentiles (box limits). The whiskers extend to the top/bottom to the maxima and minima. Data beyond the end of the whiskers are considered outliers. Left, *T. elliotii*; right, *P. monticolus*.

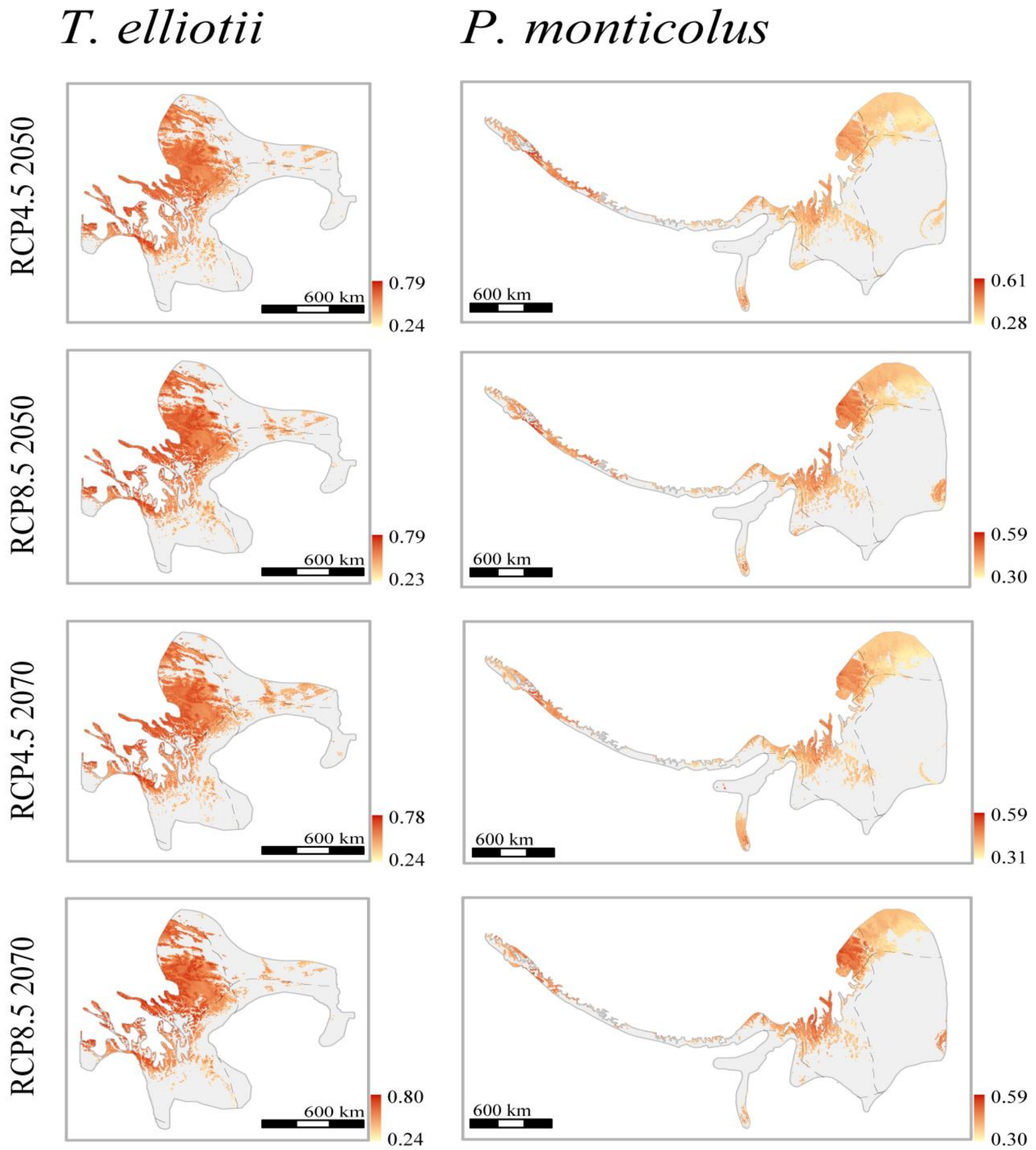

Supplementary Fig. 7. Projection of genome-niche index based on the combined estimate of genomic offset and niche suitability change under different future climate conditions (RCP4.5 2050, RCP8.5 2050, RCP4.5 2070 and RCP8.5 2070). Left, *T. elliotii*, right, *P. monticolus*. After ABC algorithm converged, we obtained optimal estimates of *nsc* α=0.558771 for *T. elliotii* and α=0.60792 for *P. monticolus* under RCP4.5 2050, α=0.52725 for *T. elliotii* and α=0.617253 for *P. monticolus* under RCP8.5 2050, α=0.548771 for *T. elliotii* and α=0.622739 for *P. monticolus* under RCP4.5 2070, and α=0.531756 for *T. elliotii* and α=0.627792 for *P. monticolus* under RCP8.5 2070.

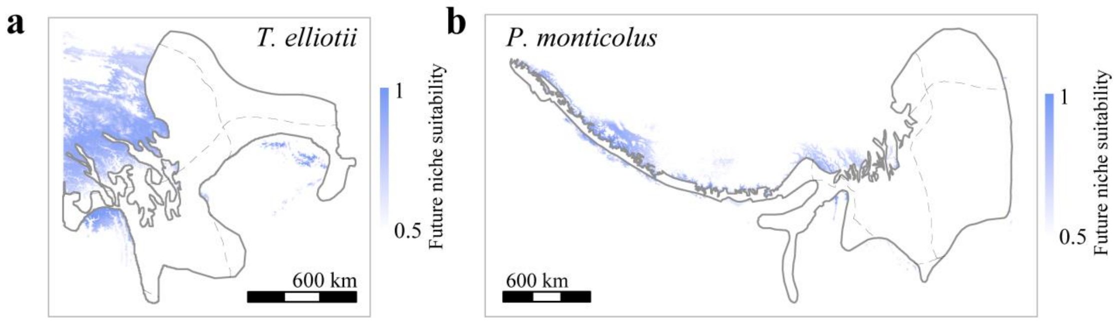

Supplementary Fig. 8. The potential niches expand out of the current distribution ranges of the two species. The large areas of the niches expanded (blue shade) for the cold-dry tolerant populations, but few for the warm-humid tolerant and warm-dry tolerant groups. Only the projection from RCP8.5 2050 is shown, as those projections under other future climatic conditions (RCP4.5 2050, RCP4.5 2070 and RCP8.5 2070) show similar results.

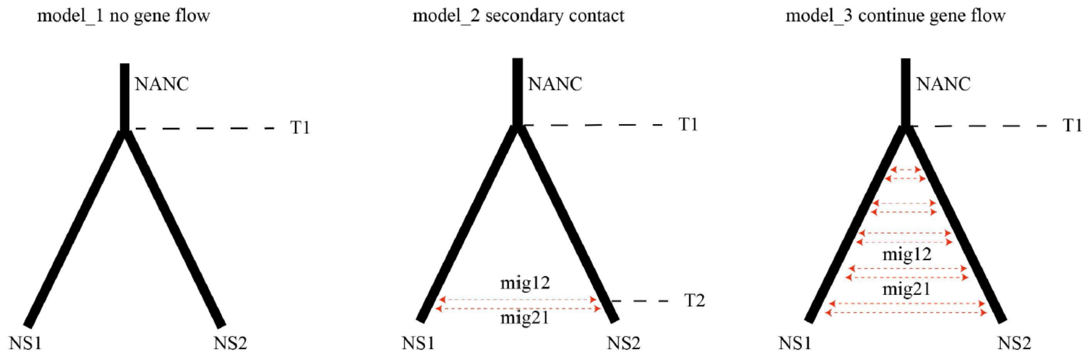

Supplementary Fig. 9. Schematics of the three demographic models testing migration probability between pairs of groups. Horizontal arrows represent the presence of gene flow. NANC, the effective population size of ancestor; NS1, the effective population size of group 1; NS2, the effective population size of group 2; , T1, split time of two groups; T2, the time having secondary gene flow between two groups; mig12 and mig21, migration probability between two groups.

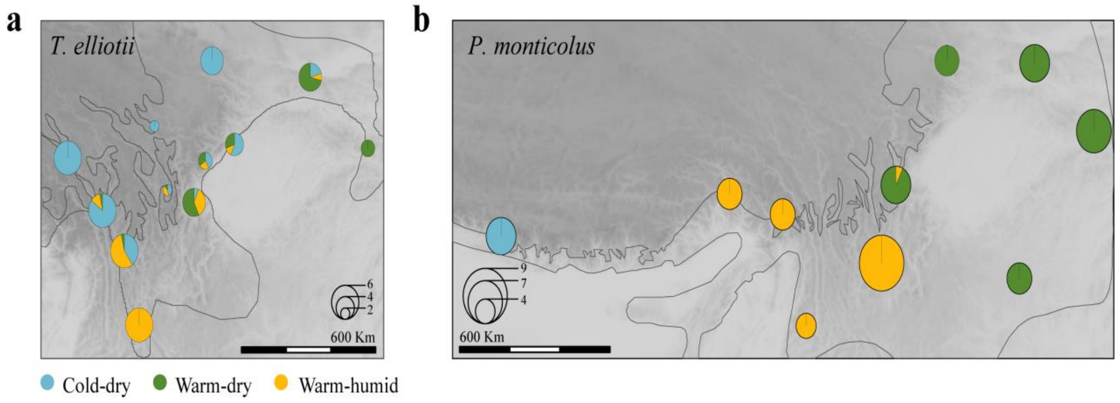

Supplementary Fig. 10. Genetic component distribution of the three groups of *T. elliotii* and *P. monticolus* along the sampling localities, based on the optimal genetic cluster *K*=3 identified by the smallest cross-validation error. Components of the cold-dry tolerant group (blue), warm-dry tolerant group (green) and warm-humid tolerant group (yellow) in each locality are shown by the pie graph. The bubble size corresponds to sample size. (a) *T. elliotii*, (b) *P. monticolus*.

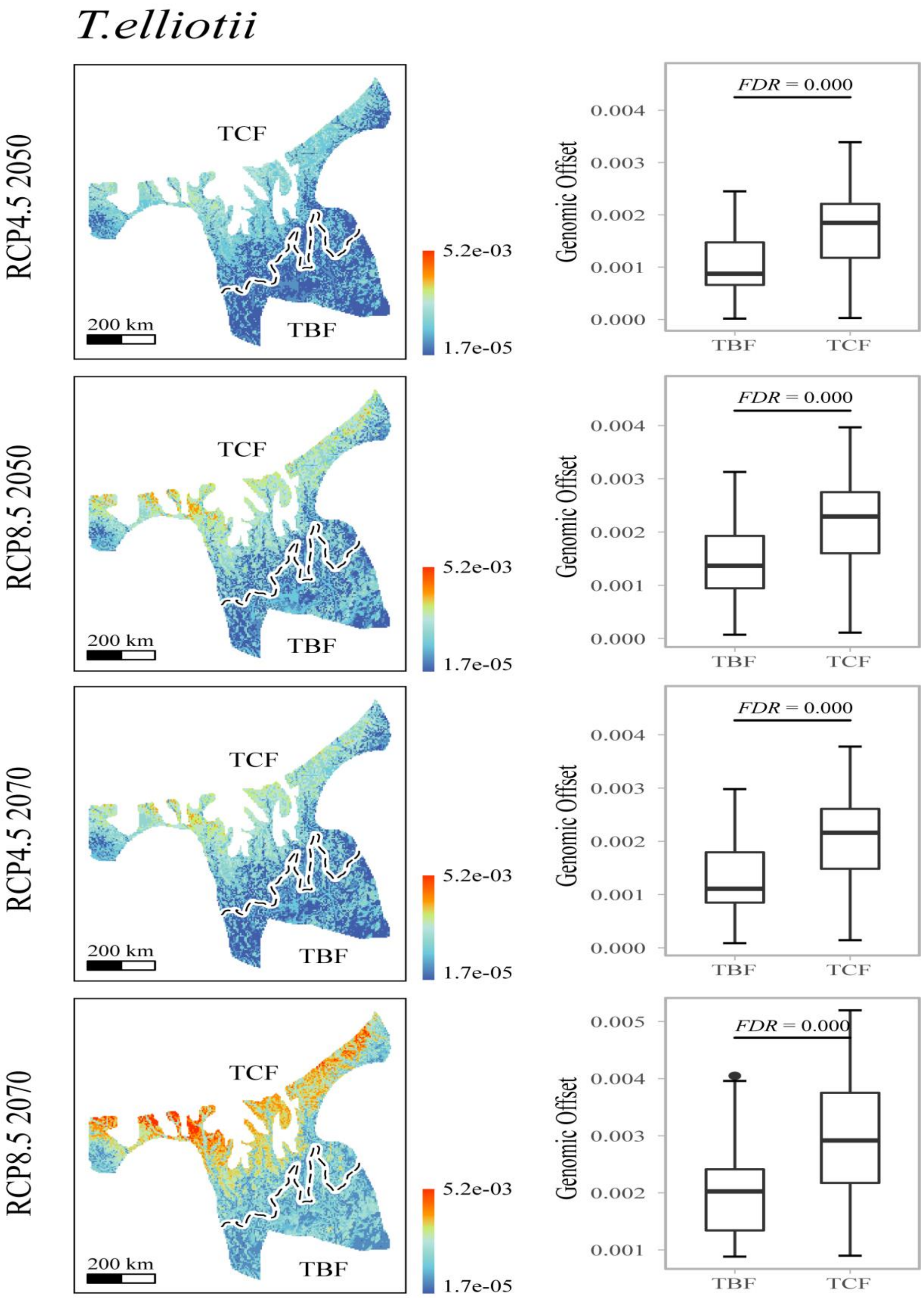

Supplementary Fig. 11. Within the *Southwest mountainous zone*, populations of *T. elliotii* in the temperate coniferous forests (TCF, *n*=9,659 grids) in the northern part show greater genomic offset than those in the tropical broadleaf forests (TBF, *n*=5,322 grids, two-tailed Wilcoxon rank sum test, P < 0.001) in the southern part. The box plots show the median (center line) and 25th-75th percentiles (box limits). The whiskers extend to the top/bottom to the maxima and minima. Data beyond the end of the whiskers are considered outliers.

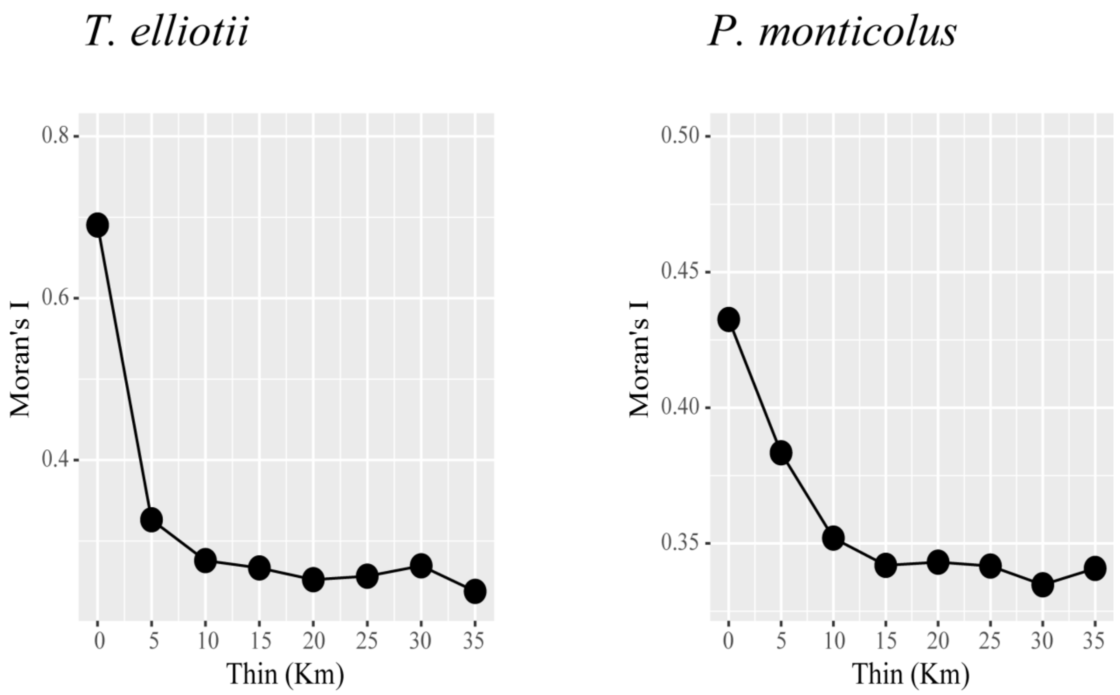

Supplementary Fig. 12. Sampling bias and autocorrelation indicated by Moran's I. Moran's I decreased and reached to a stable level at the approximately 10-km and 20-km thresholds.

Supplementary Table 1. 19 climatic variables used for genomic offset analysis and ecological niche modelling.

Climatic variables	Definition
BIO1	Annual mean temperature
BIO2	Mean diurnal range (mean of the monthly differences between maximum and minimum temperature)
BIO3	Isothermality (mean diurnal range/temperature annual range) (*100)
BIO4	Temperature seasonality (standard deviation *100)
BIO5	Max temperature of the warmest month
BIO6	Min temperature of the coldest month
BIO7	Temperature annual range (BIO5-BIO6)
BIO8	Mean temperature of the wettest quarter
BIO9	Mean temperature of the driest quarter
BIO10	Mean temperature of the warmest quarter
BIO11	Mean temperature of the coldest quarter
BIO12	Annual precipitation
BIO13	Precipitation of the wettest month
BIO14	Precipitation of the driest month
BIO15	Precipitation seasonality (Coefficient of variation)
BIO16	Precipitation of the wettest quarter
BIO17	Precipitation of the driest quarter
BIO18	Precipitation of the warmest quarter
BIO19	Precipitation of the coldest quarter

Supplementary Table 2. Rank and cumulative contributions of climatic variables in the GradientForest analysis. Top-ranked uncorrelated variables used for gradientForest analyses are marked by stars. These variables were selected by moving down the list of ranked importance for the full model and discarding variables highly correlated with a variable of higher importance (Pearson's r >0.70).

**Species**	**Code**	**Definition**	**Cumulative contribution**
T.elliotii	BIO2*	Mean diurnal range	8.07%
	BIO3	Isothermality	14.61%
	BIO10*	Mean temperature of warmest quarter	20.99%
	BIO1	Annual mean temperature	27.25%
	BIO6	Min temperature of coldest month	33.31%
	BIO8	Mean temperature of wettest quarter	39.35%
	BIO12	Annual precipitation	45.24%
	BIO16	Precipitation of wettest quarter	50.74%
	BIO11	Mean temperature of coldest quarter	56.16%
	BIO13	Precipitation of wettest month	61.31%
	BIO18	Precipitation of warmest quarter	66.23%
	BIO7*	Temperature annual range	71.01%
	BIO9	Mean temperature of driest quarter	75.78%
	BIO5	Max temperature of warmest month	80.54%
	BIO19*	Precipitation of coldest quarter	84.81%
	BIO14	Precipitation of driest month	89.08%
	BIO17	Precipitation of driest quarter	93.32%
	BIO4*	Temperature seasonality	96.99%
	BIO15	Precipitation seasonality	100.00%
P. monticolus	BIO3*	Isothermality	8.36%
	BIO4	Temperature seasonality	16.64%
	BIO18*	Precipitation of warmest quarter	24.54%
	BIO16	Precipitation of wettest quarter	32.03%
	BIO13	Precipitation of wettest month	39.01%
	BIO7	Temperature annual range	45.33%
	BIO15	Precipitation seasonality	50.76%
	BIO12	Annual precipitation	56.11%
	BIO9*	Mean temperature of driest quarter	61.25%
	BIO11	Mean temperature of coldest quarter	66.02%
	BIO2	Mean diurnal range	70.68%
	BIO19*	Precipitation of coldest quarter	74.99%
	BIO5*	Max temperature of warmest month	79.03%
	BIO6	Min temperature of coldest month	83.02%
	BIO1	Annual mean temperature	86.90%
	BIO8	Mean temperature of wettest quarter	90.71%
	BIO17	Precipitation of driest quarter	94.14%
	BIO10	Mean temperature of warmest quarter	97.52%
	BIO14	Precipitation of driest month	100.00%

Supplementary Table 3. The climate models (MPI-ESM-LR, CCSM4, CNRM-CM5-2 and MICRO-5) and emission scenarios and decades (RCP4.5 2050, RCP8.5 2050, RCP4.5 2070 and RCP8.5 2070) used in the genomic offset analysis and ecological niche modelling.

Models	Source
MPI-ESM-LR	The Max Planck Institute for Meteorology Earth System Model
CCSM4	The Community Climate System Model Version 4
CNRM-CM5-2	Centre National de Recherches Météorologiques
MICRO-5	Model for Interdisciplinary Research on Climate, ver. 3.2; http://www.ccsr.u-tokyo. ac.jp/hasumi/MIROC/
Scenarios	Description
RCP4.5(2050, 2070)	A possible radiative forcing values at 4.5 W/m2 in the year 2100
RCP8.5(2050, 2070)	A possible radiative forcing values at 8.5 W/m2 in the year 2100

Supplementary Table 4. 25 and 204 climate-associated SNPs are identified by the three genotype-climate association methods (LFMM, RAD and dbRAD). These SNPs are annotated in the coding sequence and promoter regions (5k upstream and downstream of genes) across 23 and 147 genes for *T. elliotii* and *P. monticolus*, respectively.

Species	Genic regions	SNPs	Proportion
T. elliotii	Coding sequence	2	8%
	Promoter (upstream)	17	68%
	Promoter (downstream)	6	24%
	In total	25	
P. monticolus	Coding sequence	29	14.21%
	Promoter (upstream)	95	46.57%
	Promoter (downstream)	80	39.22%
	In total	204	

Supplementary Table 5. The ecological niche modelling performances for the two species.

Species	Datasets	AUC	TSS
T.elliotii	Cold-dry tolerant group	0.928	0.75
	Warm-dry tolerant group	0.961	0.878
	Warm-humid tolerant group	0.964	0.859
P. monticolus	Cold-dry tolerant group	0.938	0.814
	Warm-dry tolerant group	0.905	0.704
	Warm-humid tolerant group	0.904	0.71

Supplementary Table 6. Modelling results of maximum likelihood population effects (MLPE) of the two species in landscape connectivity analyses. SI, habitat suitability predicted by ecological niche modelling; LC, land cover; R_m^2, marginal R^2; R_c^2, conditional R^2.

Species	Models	AIC	k	AICc	w_iAICc	ΔAICc	R_m^2	R_c^2
T. elliotii	SI	86.30	4	86.95	0.32	0.00	0.42	0.92
	LC+SI	87.97	5	88.97	0.12	2.02	0.44	0.94
	slope+SI	88.22	5	89.22	0.10	2.26	0.38	0.91
	elevation+ST	88.22	5	89.22	0.10	2.27	0.39	0.92
	Elevation	88.90	4	89.56	0.09	2.60	0.21	0.89
	Slope	90.13	4	90.78	0.05	3.83	0.10	0.88
	LC+elevation+SI	89.60	6	91.02	0.04	4.07	0.37	0.93
	LC+slope+SI	89.80	6	91.23	0.04	4.27	0.39	0.93
	LC+elevation	90.46	5	91.46	0.03	4.51	0.17	0.90
	elevation+elevation+SI	90.21	6	91.63	0.03	4.68	0.37	0.92
	slope+elevation	90.86	5	91.86	0.03	4.91	0.18	0.89
	LC+slope	92.11	5	93.11	0.01	6.16	0.09	0.89
	LC+elevation+slope+SI	91.59	7	93.52	0.01	6.57	0.38	0.94
	LC+slope+elevation	92.46	6	93.88	0.01	6.93	0.17	0.91
	LC	96.22	4	96.87	0.00	9.92	0.35	0.90
P. monticolus	elevation+SI	28.38	5	29.92	0.37	0.00	0.57	0.94
	Elevation	29.52	4	30.52	0.27	0.60	0.23	0.92
	LC+elevation+SI	29.43	6	31.64	0.16	1.71	0.59	0.94
	LC+elevation	31.27	5	32.81	0.09	2.89	0.24	0.93
	LC	32.61	4	33.61	0.06	3.69	0.33	0.95
	SI	33.43	4	34.43	0.04	4.51	0.10	0.94
	LC+SI	33.97	5	35.51	0.02	5.59	0.21	0.94

Supplementary Table 7. Model selection using Fastsimcoal v2.6 and Akaike information criterion (AIC). Bold shows the best models supported by ΔAIC and AIC's weight.

Species	Models	MaxEstLhood	AIC	ΔAIC	AIC's weight (W)
T. elliotii					
Cold-dry vs. warm-dry groups	Non gene flow model (M1)	-10816746.0	49812964.1	4853.65	0
	**Secondary gene flow model (M2)**	**-10806207.9**	**49764440.5**	**0**	**1**
	Continuous gene flow model (M3)	-10807626.4	49770970.8	6530.30	0
Cold-dry vs. warm-humid groups	Non gene flow model (M1)	-10957339.6	50460421.6	54208.6	0
	**Secondary gene flow model (M2)**	**-10945567.0**	**50406213.0**	**0**	**1**
	Continuous gene flow model (M3)	-10947247.4	50413949.2	7736.3	0
Cold-dry vs. warm-humid groups	Non gene flow model (M1)	-10910021.9	50242515.7	48885.7	0
	**Secondary gene flow model (M2)**	**-10899405.2**	**50193630.1**	**0**	**1**
	Continuous gene flow model (M3)	-10902037.3	50205749.2	12119.1	0
P. monticolus					
Cold-dry vs. warm-dry groups	Non gene flow model (M1)	-7569384.5	34858311.7	346232.9	0
	**Secondary gene flow model (M2)**	**-7494199.7**	**34512078.9**	**0**	**1**
	Continuous gene flow model (M3)	-7499413.2	34536086.7	24007.8	0
Cold-dry vs. warm-humid groups	Non gene flow model (M1)	-7410043.0	34124517.1	397440.2	0
	**Secondary gene flow model (M2)**	**-7323738.6**	**33727076.9**	**0**	**1**
	Continuous gene flow model (M3)	-7327639.9	33745040.7	17963.9	0
Cold-dry vs. warm-humid groups	Non gene flow model (M1)	-7140412.0	32882820.3	391217.2	0
	**Secondary gene flow model (M2)**	**-7055458.9**	**32491603.1**	**0**	**1**
	Continuous gene flow model (M3)	-7057596.0	32501442.4	9839.3	0

Supplementary Table 8. Parameter estimates under the best-fit demographic model (M2, secondary gene flow). All estimates assume an one-year generation time and a mutation rate of 3.3e^{-9} per site per generation. Point estimates are provided with 95% confidence intervals in parentheses. *m*, migration probabilities (or, the probability that any gene from one lineage transfers to another on a per generation basis).

Demographic model	Paramet	Description	Estimate values
T. elliotii			
Cold-dry tolerant vs. warm-dry tolerant lineages	T1	Split time between lineages	27,871 (22,639-33,103)
	T2	Admixture time	16,310 (11,197-21,423)
	MIG12	*m* from warm-dry to cold-dry lineages	1.76e^{-3} (2.02e^{-05}-3.35e^{-3})
	MIG21	*m* from cold-dry to warm-dry lineages	6.69e^{-6} (3.2e^{-06}-1.02e^{-5})
Cold-dry tolerant vs. warm-humid tolerant lineages	T1	Split time between lineages	32,165 (38,905-68,596)
	T2	Admixture time	17,524 (10,062-24,986)
	MIG12	*m* from warm-dry to cold-dry lineages	3.07e^{-3} (0-6.2e^{-3})
	MIG21	*m* from cold-dry to warm-dry lineages	7.69e^{-5} (5.99e^{-06}-1.48e^{-4})
Warm-dry tolerant vs. warm-humid tolerant lineages	T1	Split time between lineages	27,429 (19,868-34,990)
	T2	Admixture time	13,260 (8,961-17,559)
	MIG12	*m* from warm-humid to warm-dry lineages	3.08e^{-4} (9.57e^{-05}-5.2e^{-4})
	MIG21	*m* from warm-dry to warm-humid lineages	8.89e^{-6} (1.46e^{-06}-1.63e^{-5})
P. monticolus			
Cold-dry tolerant vs. warm-dry tolerant lineages	T1	Split time between lineages	85,965 (69,152-102,778)
	T2	Admixture time	12,807 (9,654-15,960)
	MIG12	*m* from warm-dry to cold-dry lineages	2.04e^{-4} (9.33e^{-05}-3.15e^{-4})
	MIG21	*m* from cold-dry to warm-dry lineages	1.06e^{-4} (7.39e^{-05}-1.38e^{-4})
Cold-dry tolerant vs. warm-humid tolerant lineages	T1	Split time between lineages	86,298 (75,803-96,793)
	T2	Admixture time	11,474 (10,023-12,924)
	MIG12	*m* from warm-dry to cold-dry lineages	7.56e^{-5} (6.29e^{-05}-8.83e^{-5})
	MIG21	*m* from cold-dry to warm-dry lineages	1.10e^{-4} (8.30e^{-05}-1.37e^{-4})
Warm-dry tolerant vs. warm-humid tolerant lineages	T1	Split time between lineages	47,601(34,921-60,283)
	T2	Admixture time	16,839 (10,941-22,737)
	MIG12	*m* from warm-humid to warm-dry lineages	1.35e^{-3} (8.46e^{-04}-1.86e^{-4})
	MIG21	*m* from warm-dry to warm-humid lineages	2.34e^{-4} (1.08e^{-4}-3.61e^{-4})

Supplementary Table 9. The climatic variables used for ecological niche modelling.

Species	Group	Climatic variables used in ecological niche modelling
T. elliotii	Cold-dry tolerant group	BIO2, BIO3, BIO8, BIO15
	Warm-dry tolerant group	BIO2, BIO3, BIO8, BIO13, BIO19
	Warm-humid group	BIO2, BIO4, BIO14, BIO15, BIO18
P. monticolus	Cold-dry tolerant group	BIO2, BIO9, BIO14, BIO15, BIO18
	Warm-dry tolerant group	BIO3, BIO4, BIO8, BIO13, BIO15
	Warm-humid group	BIO2, BIO3, BIO4, BIO8, BIO15, BIO18, BIO19

Supplementary Table 10. Sensitivity analyses of different assignments of resistance costs to habitat types in the land cover. R_m^2, marginal R^2; R_c^2, conditional R^2.

Resistance costs	R_m^2/R_c^2 of *T.elliotii*	R_m^2/R_c^2 of *P. monticolus*
Forest=1<br>Shrublands=1<br>Grasslands=1<br>Savannas=3<br>Croplands=5<br>Barren or Sparsely Vegetated=7<br>Water=10<br>Urban and Built-Up=10	0.355/0.904	0.325/0.951
Forest=1<br>Shrublands=1<br>Grasslands=1<br>Savannas=4<br>Croplands=5<br>Barren or Sparsely Vegetated=9<br>Water=10<br>Urban and Built-Up=10	0.354/0.904	0.321/0.952
Forest=1<br>Shrublands=2<br>Grasslands=1<br>Savannas=4<br>Croplands=5<br>Barren or Sparsely Vegetated=8<br>Water=10<br>Urban and Built-Up=10	0.344/0.902	0.315/0.947
Forest=10<br>Shrublands=10<br>Grasslands=10<br>Savannas=7<br>Croplands=5<br>Barren or Sparsely Vegetated=3<br>Water=1<br>Urban and Built-Up=1	0.157/0.888	0.05/0.934
