# Supplementary material for: The combination of genomic offset and niche modelling provides insights into climate change-driven vulnerability
Source: Nat Commun. 2022 Aug 16;13:4821. doi: 10.1038/s41467-022-32546-z (PMC9381542; doi:10.1038/s41467-022-32546-z)
Supplement: Supplementary file 3 — Description to Additional Supplementary Information [file 41467_2022_32546_MOESM3_ESM.docx]

Description of Additional Supplementary Files

File Name: Supplementary Data 1
Description: Sampling of *T. elliotii* and *P. monticolus* used in this study.

File Name: Supplementary Data 2
Description: Re-sequencing statistic of the two species used in this study.
