# Supplementary material for: The combination of genomic offset and niche modelling provides insights into climate change-driven vulnerability
Source: Nat Commun. 2022 Aug 16;13:4821. doi: 10.1038/s41467-022-32546-z (PMC9381542; doi:10.1038/s41467-022-32546-z)
Supplement: Supplementary file 6 — Reporting Summary [file 41467_2022_32546_MOESM6_ESM.pdf]

nature portfolio

Corresponding author(s): Yanhua Qu; Fumin Lei

Last updated by author(s): Jul 17, 2022

Reporting Summary

Nature Portfolio wishes to improve the reproducibility of the work that we publish. This form provides structure for consistency and transparency in reporting. For further information on Nature Portfolio policies, see our Editorial Policies and the Editorial Policy Checklist.

Statistics

For all statistical analyses, confirm that the following items are present in the figure legend, table legend, main text, or Methods section.

n/a	Confirmed	
☐	☒	The exact sample size (*n*) for each experimental group/condition, given as a discrete number and unit of measurement
☐	☒	A statement on whether measurements were taken from distinct samples or whether the same sample was measured repeatedly
☐	☒	The statistical test(s) used AND whether they are one- or two-sided<br>*Only common tests should be described solely by name; describe more complex techniques in the Methods section.*
☐	☒	A description of all covariates tested
☐	☒	A description of any assumptions or corrections, such as tests of normality and adjustment for multiple comparisons
☐	☒	A full description of the statistical parameters including central tendency (e.g. means) or other basic estimates (e.g. regression coefficient) AND variation (e.g. standard deviation) or associated estimates of uncertainty (e.g. confidence intervals)
☐	☒	For null hypothesis testing, the test statistic (e.g. *F*, *t*, *r*) with confidence intervals, effect sizes, degrees of freedom and *P* value noted<br>*Give P values as exact values whenever suitable.*
☒	☐	For Bayesian analysis, information on the choice of priors and Markov chain Monte Carlo settings
☒	☐	For hierarchical and complex designs, identification of the appropriate level for tests and full reporting of outcomes
☐	☒	Estimates of effect sizes (e.g. Cohen's *d*, Pearson's *r*), indicating how they were calculated

Our web collection on statistics for biologists contains articles on many of the points above.

Software and code

Policy information about availability of computer code

Data collection	De novo genome: sequencing was carried out on BGI-seq 500 platform with PE 150; cleaned raw sequencing data using SOAPfilter v2.2; assembled the genome using Supernova v2.0.1 and filled the intra-scaffold gaps using GapCloser v1.12; estimated genome completeness using BUSCO v3.0.2; annotated protein-coding genes using TABASTN v2.2.26; genBlastA v1.0.4; Genewise v2.4.1; Muscle v3.8.31.<br>Resequencing data were sequenced on the Illumina sequencing platform (NovaSeq 6000) at Berry Genomics Corporation (Beijing, China); cleaning and evaluation: Trimmomatic v0.32 (Bolger et al. 2014), FastQC v0.11.5 (Andrews, http://bioinformatics.babraham.ac.uk/projects/fastqc/)
Data analysis	Mapping reads using BWA v0.7.17; calling SNPs using HaplotypeCaller function from GATK v3.7.0 and Samtools v1.3.1., VCFtools v0.1.12b; estimating genomic offsets using R packages gradientForest and GDM; genotype-climate association analyses, LFMM, RAD and dbRAD; Ecological niche modeling, Biomod2 and ENMwizard; integrating niche suitability and genomic offset, R package ABCoptim; landscape genetic analysis, Circuitscape; estimating population pairwise FST matrix, R package diveRsity

For manuscripts utilizing custom algorithms or software that are central to the research but not yet described in published literature, software must be made available to editors and reviewers. We strongly encourage code deposition in a community repository (e.g. GitHub). See the Nature Portfolio guidelines for submitting code & software for further information.

Data

Policy information about availability of data

All manuscripts must include a data availability statement. This statement should provide the following information, where applicable:

- Accession codes, unique identifiers, or web links for publicly available datasets
- A description of any restrictions on data availability
- For clinical datasets or third party data, please ensure that the statement adheres to our policy

The genome assembly and sequencing data of the de novo sequenced individual of T. elliotii have been deposited to National Genomics Data center (https://db.cngb.org/) under BioProject accession CNP0003256 (https://db.cngb.org/search/project/CNP0003256/) and NCBI with BioProject accession PRJNA860040 (https://www.ncbi.nlm.nih.gov/bioproject/?term=PRJNA860040). The resequencing data from P. monticolus and T. elliotii have been deposited in the National Genomics Data center (https://db.cngb.org/) under the accession number CNP0002314 (https://db.cngb.org/search/?q=CNP0002314) and CNP0002315 (https://db.cngb.org/search/?q=CNP0002315), respectively. VCF datasets, location records used in ecological niche modelling, climatic data used in genotype-climate-association, outputs of ecological niche modelling and genomic offset analyses are available in Dryad (doi.org/10.5061/dryad.brv15dvb5).

Human research participants

Policy information about studies involving human research participants and Sex and Gender in Research.

Reporting on sex and gender	N/A
Population characteristics	N/A
Recruitment	N/A
Ethics oversight	N/A

Note that full information on the approval of the study protocol must also be provided in the manuscript.

Field-specific reporting

Please select the one below that is the best fit for your research. If you are not sure, read the appropriate sections before making your selection.

☐ Life sciences ☐ Behavioural & social sciences ☒ Ecological, evolutionary & environmental sciences

For a reference copy of the document with all sections, see nature.com/documents/nr-reporting-summary-flat.pdf

Ecological, evolutionary & environmental sciences study design

All studies must disclose on these points even when the disclosure is negative.

Study description	Incorporate intraspecific variation in genotype-climate association to estimate genomic offset and niche suitability to predict climate-change driven vulnerability in two mountainous birds in the Sino-Himalayan Mountains, a temperate biodiversity hotpot.
Research sample	Sample were collected from 12 and 10 localities for T. elliotii and P. monticolus covered the main distribution ranges of the Sino-Himalayan Mountains. Details on all resequenced individuals including species, location, collection data, coordinates, elevation and sex (when known) are summarized in Supplementary Table 1. We referred each sampling locality as a population here.
Sampling strategy	We resequenced 55 and 58 individuals from 12 and 10 sampling localities for T. elliotii and P. monticolus, respectively. Each locality includes 2-9 individuals. This sampling was designed to maximumly cover the main distribution ranges of two species in the Sino-Himalayan Mountains, including different eco-regions in Sino-Himalayan Mountains. However, our study areas are in highly heterogeneous mountains, which present a great challenge for obtaining samples. Although our study, to our knowledge, represents the densest bird sampling in this region, we could only include two or three individuals of T. elliotii for three localities (i.e., Rangtang, Mangkang and Wulong). Given this small sampling size, we implemented a deep-sequencing strategy because low precision in statistical inference due to the low sample size can be offset by using a large number of SNPs in the analyses. In addition, we have also conducted a parallel genomic offset analysis using a distance-based method (i.e., FST), generalized dissimilarity modelling, to estimate the relationship between genetic variation and climatic variables. It has been shown that FST analysis provides reliable estimates even for populations with small sample sizes (i.e., two individuals) given a large number of SNPs (i.e., >1500).
Data collection	Birds were collected using mist nets, which is the ususal method for collecting in the field. Pen and paper were used to note details. Birds were collected by Xu Luo, Gang Song, Fumin Lei and collaborators.
Timing and spatial scale	The samples from this study are collected in a time period of 15 years (between 2000-2020) in west and south part of China
Data exclusions	Sequencing reads did not pass filtering conditions were excluded from genomic study. Distribution records used in the ecological niche modeling were filtered by a 10-km threshold. The redundant records were removed and only one record in every ten

	kilometers was kept.
Reproducibility	All methods are thoroughly described to allow reproducibility. We also provided all codes used in this study in DRYAD (doi.org/10.5061/dryad.brv15dvb5) that allow to repeat analyses.
Randomization	We implemented the population genetic structure analysis using genotypes associated with climatic variables, and then divided samples into three populations according to the optimal K value identified by population genetic structure analysis (k=3). As we aim to find whether there is a intraspecific variation in response to climate changes, dividing samples into different climate-tolerant populations provides a reasonable grouping to compare population-specific responses.
Blinding	Blinding was not relevant to our study because we aim to find climate-associated genetic variation. In this case, selection sampling localities across different climatic zone is particular important.

Did the study involve field work? ☐ Yes ☒ No

Reporting for specific materials, systems and methods

We require information from authors about some types of materials, experimental systems and methods used in many studies. Here, indicate whether each material, system or method listed is relevant to your study. If you are not sure if a list item applies to your research, read the appropriate section before selecting a response.

Materials & experimental systems

n/a	Involved in the study
☒	☐ Antibodies
☒	☐ Eukaryotic cell lines
☒	☐ Palaeontology and archaeology
☐	☒ Animals and other organisms
☒	☐ Clinical data
☒	☐ Dual use research of concern

Methods

n/a	Involved in the study
☒	☐ ChIP-seq
☒	☐ Flow cytometry
☒	☐ MRI-based neuroimaging

Animals and other research organisms

Policy information about studies involving animals; ARRIVE guidelines recommended for reporting animal research, and Sex and Gender in Research

Laboratory animals	This study did not involve laboratory animals
Wild animals	Samples were collected from the field by mist net during scientific explorations over 15 years. The birds were killed to obtain genetic materials and museum study specimens. The birds were killed in a humane way by cervical dislocation to ensure that the animals did not suffer unnecessarily. The Zoological Museum of Institute of Zoology has the authority for specimen collecting and exemption of export/import of samples for scientific purposes (No. 1999/84, provided by Article VII from CITES).
Reporting on sex	Sex was not considered in this study. We regarded make and female responded similarly to the climate change.
Field-collected samples	No live birds were transported. The genetic materials of the birds were stored in liquid nitrogen in the filed. When they exported into museum, they are stored in the deep freezer (-80 Celsius degree) in the Zoological museum of Institute of Zoology, Chinese Academy of Sciences.
Ethics oversight	Tissue collection procedures also conform to the regulations of the Animal Experimental and Medical Ethics Committee of the Institute of Zoology, Chinese Academy of Sciences.

Note that full information on the approval of the study protocol must also be provided in the manuscript.
